# *Glechoma curviflora* Volatile Oil from Palestine: Chemical Composition and Neuroprotective, Antimicrobial, and Cyclooxygenase Inhibitory Activities

**DOI:** 10.1155/2020/4195272

**Published:** 2020-11-23

**Authors:** Nawaf Al-Maharik, Nidal Jaradat, Mohammad Qneibi, Murad N. Abualhasan, Nour Emwas

**Affiliations:** ^1^Department of Chemistry, Faculty of Sciences, An-Najah National University, Nablus, State of Palestine; ^2^Department of Pharmacy, Faculty of Medicine and Health Sciences, An-Najah National University, Nablus, State of Palestine; ^3^Department of Biomedical Sciences, Faculty of Medicine and Health Sciences, An-Najah National University, Nablus, State of Palestine

## Abstract

The rise of the emergence of microbial resistance of antibiotics, the dangerous side effects of nonsteroidal anti-inflammatory drugs, and noncompetent medications of Alzheimer's, Parkinson's, and other neurodegenerative diseases prompt scientists to search for phytochemicals that could be utilized in the remedy of lethal diseases. *Glechoma curviflora* (Boiss.) Kuntze (*Nepeta curviflora*) is a medicinal herb growing in the eastern parts of the Mediterranean Sea Basin and is widely consumed as a tea. The leaves of this plant have been traditionally used for the treatment of various infectious diseases. The current research was designed to identify the chemical composition of *Glechoma curviflora* (Boiss.) essential oil (EO) and to assess its antibacterial, antifungal, and cyclooxygenase inhibitory activities and the biophysical gating effect on AMPA receptors. Twenty phytochemicals were identified from *G. curviflora* leaves and flowers EO amounting to almost 100% of the total constituents using GC-MS technique, of which 1,6-dimethylspiro[4.5]decane (27.51%) **1**, caryophyllene oxide (20.08%) **2**, and *β*-caryophyllene (18.28%) **3** were the main constituents. The biophysical properties' effect from the plant extract on various AMPA-type receptors expressed in Human Embryonic Kidney (HEK293) cells was assessed by exploiting the whole-cell patch-clamp technique. Microdilution assay was adopted for assessing the antimicrobial property against eight virulent microbial strains whilst the cyclooxygenase inhibition effect was accomplished utilizing COX inhibitory screening colorimetric assay *G. curviflora* EO displayed potent activity against *P. aeruginosa* (MIC = 1.25 *μ*g/mL), *S. sonnei* (MIC = 3.12 *μ*g/mL), and *E. coli* (MIC = 1.25 *μ*g/mL), compared with ciprofloxacin (positive control) and potent antibacterial activity against *S. aureus*, MRSA, *S. sonnei*, *E. coli*, and *P. aeruginosa* compared to Ampicillin (2nd positive control). It also showed anti-*Candida* (MIC = 6.25 *μ*g/mL) and antimold (MIC = 3.125 *μ*g/mL) activities compared with fluconazole (antifungal positive control). Likewise, our results showed an inhibition and biophysical impact of *G*. curviflora on all AMPARs subunits.

## 1. Introduction

The search for medications and dietary supplements derived from plants has accelerated in the last two decades. Botanists, phytochemists, microbiologists, and pharmacologists are combing the Earth searching for natural products that have therapeutic potentials [1]. Essential oils (EOs) are valuable natural plant products known for their therapeutic importance and have been widely employed in cosmetics, perfumes, foods, flavoring agents, and beverages industries [2].

Postsynaptic membranes expressing cation glutamatergic AMPA receptors (AMPARs) are implicated in various plasticity and synaptic transmissions. In the mammalian brain, the postsynaptic AMPARs are fundamentally essential in facilitating the predominantly fast excitatory transmission. Overactivated AMPARs trigger excitotoxicity, involved in numerous neurodegenerative severe illnesses such as AIDS encephalitis, Amyotrophic Lateral Sclerosis (ALS), Parkinson's, epilepsy, Huntington's, and other neurological disorders [3].

Microbial infections due to drug-resistant strains turn out to be one of the most serious intimidations to public health. Numerous health care systems and research centers, especially in developed countries, are relentlessly searching for alternative therapies to cure such infections. Herbal medicines are acknowledged to prevent infections as they are considered a better alternative to artificial drugs due to drug-resistant pathogens [4].

Cyclooxygenase (COX-1 or COX-2) inhibitors, classified as NSAIDs-nonsteroidal anti-inflammatory drugs, are considered to be the most common medications for inflammatory diseases due to their effectiveness in the treatment of fever, redness, pain, and edema. Most of the used NSAIDs are associated with harmful side effects, including gastrointestinal ulcers, renal failure, and hepatic dysfunction [5, 6].

*Glechoma curviflora* (Boiss.) Kuntze (*Nepeta curviflora*) is a perennial aromatic herb growing in the eastern parts of the Mediterranean Sea Basin, which is widely consumed as a tea in Lebanon, Syria, Jordan, Turkey, and Palestine [7]. It is utilized for the treatment of diarrhea, bronchitis, fever, flu, colic, cough, colds, angina pectoris, and tachycardia. It is also used as antiseptic, diuretic, carminative, and sedative [8]. *G. curviflora* is utilized in folk medicine for curing different infectious and Alzheimer's disease and as an antipyretic agent [9]. The current study aims to identify and quantify the chemical components and to evaluate the antimicrobial, cyclooxygenase inhibitory, and neuroprotective effects of *G. curviflora* EO.

## 2. Material and Methods

### 2.1. Plant Collection and Essential Oil Extraction

The flowers and leaves of the *G. curviflora* plant were compiled from the Birzeit mountains in Palestine in May 2019. The taxonomical characterization was executed by Dr. Nidal Jaradat a pharmacognosist. The dried herbarium was kept at the Herbal Products Laboratory labeled as Pharm-PCT-1633 obtained from the voucher specimen code. After washing with distilled water, the leaves and flowers were left to dry in the shadow at a 25 ± 2°C temperature and 55 ± 5 RH humidity for two weeks then powdered using a mechanical grinder and stored in paper bags [10]. The *G. curviflora* EO was extracted utilizing the Ultrasonic-Microwave apparatus according to the literature procedure [11]. A 1 L round-bottom flask containing 50 g of the dried plant powder and 0.5 L of distilled water was positioned in the apparatus and connected to the Clevenger apparatus. The microwave power was adjusted at 800 W and the extraction was executed at 90°C for 20 min, where the same process was triplicated. The attained EO was dried using calcium chloride and stored at 2–8°C. The dried plant sample provided 0.905 g of a colorless oil in 1.81% yield average of the isolated EO.

### 2.2. GC-MS Qualitative and Quantitative Characterization of *G. curviflora* EO Composition

The identification of *G. curviflora* EO components was accomplished using PerkinElmer Elite-5-MS fused-silica capillary column (0.25 mm × 30 m, film thickness 0.25 *μ*m), where helium was set at 1.1 mL/min flow rate. The temperature of the injector was adjusted at 250°C with an initial temperature of 50°C, initial hold 5 min, and ramp 4.0°C/min to 280°C. The total running time was 62.50 min and the solvent delay was from 0 to 4.0 min. MS scan time was from 4 to 62.5 min, covering mass range 50.00 to 300.00 m/z. To cover a mass range of 50.00 to 300.00 m/z, the corresponding MS scan time range was 4 to 62.5 min. Comparing the mass spectra of the *G. curviflora* EO to the reference spectra in the MS data Center of the National Institute of Standards and Technology, we identified the plant's components. Finally, by matching their Kovats retention indices with the values reported in the literature, the *G. curviflora* EO components were quantified [12, 13].

### 2.3. Neuroprotective Effect Assessment

Previous work from our laboratory provides a methodology for the DNA preparation, cDNA transient transfection, and cell culturing of HEK293 to express AMPARs (of the flip isoform) of different subunits [14–16]. Two days after the chemical mediated transfection procedure, we proceeded to assess the electrophysiological recordings of the green fluorescent protein cotransfected cells. Prior preparations included the replating of the cells on coverslips layered with laminin. The cells exhibiting a strong fluorescent image were selected to proceed *via* gigaseal. The 2–4 MΩ resistance patch electrodes were produced utilizing borosilicate glass. An integrated patch amplifier IPA (Sutter Instruments, Novato, CA, and SutterPatch Software v. 1.1.1 to digitize membrane currents for a short period) was employed to perform the whole-cell patch-clamp technique. The IPA was set at 22°C, the sampling frequency at 10 kHz, and the low-pass filter set at 2 kHz, using a membrane potential of −60 mV. The extracellular solution composed of NaCl (0.15 M), KCl (2.8 mM), MgCl_2_ (0.5 mM), CaCl_2_ (2.0 mM), and NaOH that was attuned to a 7.4 pH. The pipette solution comprised of CsF (0.11 M), CsCl (30 mM), 4 NaCl (4 mM), 0.5 CaCl_2_ (0.5 mM), 10 Trypsin EDTA (10 mM) solution B (0.25%), EDTA (0.05%), and 10 HEPES (10 mM) which was adjusted to pH 7.2 with CsOH was prepared. A double-barrel glass (theta tube), set at high-speed piezo solution switcher (Automate Scientific, Berkeley, CA), was employed for continuous wash of the cell from one opening whereas the other provides the currently tested compound to the cell. Pulses per 500 ms resulted in a 10–90% solution exchange whereby the velocity of this rate is calculated from the removal of the patch from the electrode. Amplitude was induced due to the ionic strength differences from different solutions. The desensitization was calculated after applying glutamate (10 mM) for 500 ms while deactivation was applied for 20 ms. The total number of trials per experiment was 5 cells to calculate the mean of inhibition, desensitization, and deactivation. The control was recordings for the biophysical properties of glutamate alone; the recordings before and after application of treatment must be indistinguishable to authenticate the effect of EO on the cell and confirm the health of the cell.

### 2.4. Antimicrobial Activity

#### 2.4.1. Microbial Isolates

The tested microbial isolates including fungal strains and bacterial strains were provided by the American Type Culture Collection in addition to a selected multidrug resistance strain which was isolated at clinical locations in our region. The isolates comprised of three Gram-positive strains—*Enterococcus faecium* (ATCC 700221), Methicillin-Resistant *Staphylococcus aureus* (MRSA), and *Staphylococcus aureus* (ATCC 25923)—and three Gram-negative strains—*Shigella sonnei* (ATCC 25931), *Pseudomonas aeruginosa* (ATCC 27853), and *Escherichia coli* (ATCC 25922). The fungal isolates were *Epidermophyton floccosum* (ATCC 10231) and *Candida albicans* (ATCC 90028).

#### 2.4.2. Antimicrobial Test

The well-diffusion assay was used for screening of the antimicrobial activity of *G. curviflora* EO. The bacterial suspension was formulated by the addition of selected colonies from agar culture to a test tube containing 5 mL of nutrient broth. Then, the turbidity of the sample was compared to that of McFarland nephelometer tube no. 0.5 (1.5 × 10^8^ CFU/ml). A 1 mL of the suspension was added to 2 mL of nutrient broth (0.5 × 10^8^ CFU/ml). *G. curviflora* EO underwent a sequential broth dilution technique to evaluate their minimum inhibitory concentration (MIC) for all tested microbial strains.

#### 2.4.3. Determination of MIC

Micro broth dilution assay was utilized to evaluate the antimicrobial activity of *G. curviflora* EO. A 100 *μ*L of Mueller–Hinton broth was placed in each well of the microtitration plate, followed by the addition of 100 *μ*L of a suspension of EO in 2% aqueous DMSO (50 *μ*g/mL) into the first well which was mixed using the micropipette. 100 *μ*L from the first well was transferred to the next well. This process was replicated for the other 9 wells unlike the last well (#11) that was labeled as a negative control for bacterial growth. The remaining solution from the 100 *μ*L was discharged after mixing, since the EO was not added to well number 12 that was labeled as the positive control. Thereafter, 1 *μ*L of a bacterial suspension (from 5 × 10^7^ CFU/mL bacterial suspension) was added to all wells excluding the negative control well #11. In well number 12, the inoculated bacteria should grow because it does not contain the EO. After 24 incubation at 35°C, the lowest concentration of *G. curviflora* EO that caused inhibition of the evident bacterial growth was identified as the MIC. *G. curviflora* EO was screened twice in each run [17, 18]. The 2% DMSO solution was used as a positive control for every microbe separately to check the effect on each one (antimicrobial activity for DMSO was considered).

#### 2.4.4. Determination of the Antiyeast Activity of *G. curviflora* EO

The MIC of the studied EO against *Candida albicans* was assessed employing broth microdilution assay as described in the literature [19, 20], where the MHB was replaced with RPMI1640.

*(1) RPMI1640 Preparation*. RPMI powder (1.04 g) was dissolved into 90 mL of sterile distilled water. Afterward, MOP (3.456 g) was added to the solution. To adjust the pH of the solution to 7, a 1 M NaOH aqueous solution was added. Finally, to reach a volume of 100 mL, distilled and sterilized water was added to make up the difference. Using a 0.45 *μ*m syringe filter, the solution was sterilized at room temperature.

*(2) Broth Microdilution*. The first well consisted of 100 *μ*L of RPMI, 1640 broth media, and 100 *μ*L of *G. curviflora* EO homogenously mixed while the second well only contained 100 *μ*L of RPMI and 1640 broth media. From the first well, 100 *μ*L was extracted and supplemented to the second well. This process was replicated for the 10 following wells yet leaving the last well as a positive control with only RPMI1640 broth. The *G. curviflora* EO at concentrations of 0.065 to 55 *μ*g/mL was dispensed in the microwells to assess its antifungal properties.

*(3) Inoculum Preparation*. Sabouraud dextrose agar was used to subculture Candida albicans which was set at 37°C. After 24 h of subculturing, five colonies were added to 5 mL of 8.5% saline. To obtain a yeast concentration of 1 × 10^6^ to 5 × 10^6^ CFU/mL, we accustomed 0.5 as the cell density by McFarland's standard where the absorbance was set at 625 nm of 0.08 to 0.1. To achieve 1 × 10^3^ to 5 × 10^3^ CFU/mL, the suspension was diluted to 1:50 and then 1:20 using broth medium (RPMI1640 broth), of which 100 *μ*L to each well except that of the negative control of yeast growth (well #11). After 48 h incubation of the inoculated plates at 35°C for 48 h, a 0.5–2.5 × 10^3^ colony-forming units (CFU)/mL concentration of microbial cells was obtained.

### 2.5. Antimold Activity

The agar dilution procedure was utilized to assess the anti-*Epidermophyton floccosum* mold activity for *G. curviflora* EO [19, 21]. The autoclave sterilized Sabouraud dextrose agar (SDA) was placed in tubes and kept in a water bath at 40°C to dilute *G. curviflora* EO. The range of concentration of the prepared *G. curviflora* EO used ranged from 0.025 to 25 *μ*g/mL. At a slanted position, the tubes were left to solidify at a 25°C temperature. After the preparation of suspension from the fresh culture of *E. floccosum* having similar turbidity to that of 0.5 McFarland standard, 20 *μ*L of it was added to each of the tubes. The positive control groups to mold consisted of SDA alone tubes. After 10 days of incubation at room temperature, the results were collected by identifying the concentration that ultimately inhibited the growth of *E. floccosum* which was labeled as the minimum inhibitory concentration.

### 2.6. Cyclooxygenase Inhibitory Activity

The EO effect on COX-1 (ovine) and COX-2 (human recombinant) enzymes was determined employing the COX inhibitor screening assay kit no. 560131 of Cayman Chemical (USA). The yellow product of this enzymatic reaction was assessed using a UV-Visible-spectrophotometer (Janeway, 7300, UK) in a Microplate Reader (Bio-Rad, A112, Japan) at 415 nm. The inhibitory assays were conducted at EO concentrations of 0.25 and 0.5 *μ*g/mL with a commercial anti-inflammatory drug (celecoxib). The cyclooxygenase inhibitory activity of the tested product was assessed and calculated from the concentration–inhibition response curve *via* regression analysis to obtain an inhibited percentage of PGE2. The tested EO concentration resulted in 50% inhibition (IC_50_) in the formation of PGE2 *via* COX-1 and COX-2 enzymes.

### 2.7. Data Analysis

The data obtained from the cyclooxygenase inhibitory assay were displayed as means ± SD. *t*-test was performed to identify and quantify the chemical components while one-way ANOVA was utilized for electrophysiology recordings. The significant differences were considered at *p* ≤ 0.05 for the cyclooxygenase inhibitory assay, while that for the electrophysiology section was considered at three levels: 0.05, 0.01, and 0.001. At 50% (IC_50_), the inhibitory concentration was calculated from BioDataFit HTP 1.2 (USA). Data obtained from whole-cell current recordings were expressed as means ± SD of *n* = 5 for the number of trials, performed at −60 mV, pH 7.4 at 22°C, and analyzed using Wave Metrics, Inc. Igor Pro7.

## 3. Results

### 3.1. GC-MS Characterization of *G. curviflora* EO

**Figure 1:**
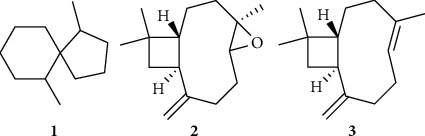
The structure of the major three components in *G. curviflora* EO.

**Figure 2:**
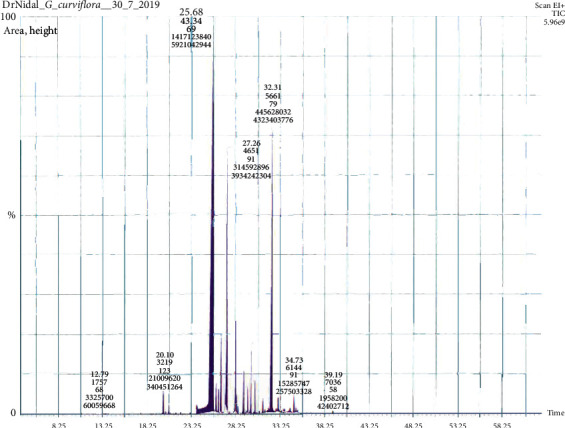
*G. curviflora* EO GC-MS chromatogram.

GC-MS analysis of the EO of *G. curviflora* growing in Palestine revealed that 1,6-dimethylspiro[4.5]decane **1** (27.51%), caryophyllene oxide **2** (20.08%), and *β*-caryophyllene **3** (18.28%) were the major constituents (Figure 1, Table 1). Twenty compounds amounting to about 100% of total EO have been identified, where the sesquiterpene hydrocarbon, spiroalkane, and oxide sesquiterpenoid groups were the major groups. The phytochemical composition, retention index (RI), and retention time (RT) with their concentration (%) are presented in Table 1 and the GC-MS chromatogram (Figure 2).

### 3.2. Influence of *G. curviflora* EO on the Biophysical Gating Properties of AMPARs

**Figure 3:**
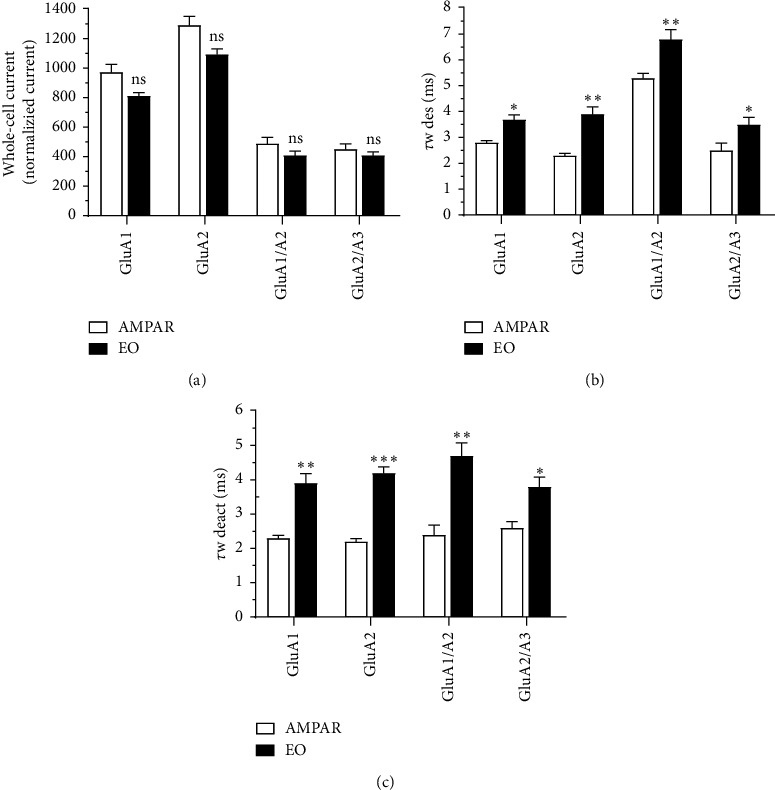
Effect of *G. curviflora* EO on AMPARs. (a) Whole-cell current recording of AMPAR subtypes with and without *G. curviflora* EO treatment. The effect of 80 *μ*M of *G. curviflora* EO (black) and 10 mM of glutamate alone (white) on the amplitude generated by different AMPARs. (b) The destination rate of various AMPARs before EO treatment (glutamate alone) vs. after treatment (glutamate + EO). Desensitization *via* the weighted time constant calculated by the pulses per 500 ms on the response of Glu alone and Glu + EO. (c) Pulses per 20 ms on the response of Glu alone and Glu + EO to calculate the deactivation. All trials performed on AMPAR expressing HEK293 cells, where the number of patch whole-cells, *n* = 5. One-way ANOVA with the following significance presented the data displayed in the figure: ^*∗*^ < 0.05; ^*∗∗*^ < 0.01; and ^*∗∗∗*^ < 0.001; ns: not significant.

The *G. curviflora* EO was evaluated for neuroprotective properties against AMPAR-current induction, deactivation, and desensitization rates. The amplitude generated by different AMPA-type receptors was recorded *via* whole-cell patch technique on HEK293 AMPAR expressing cells. Using glutamate alone, the current produced (*A*) was then compared to the induced current obtained from glutamate plus EO amplitude (*A*_*I*_) for calculating any significant inhibition solely from the extract as shown in Figure 3. Preceding the compound treatment, HEK293 cells were normalized for the control *via* 500 ms intervals of agonist; the initially acquired amplitude of GluA1, GluA2, GluA1/2, and GluA2/3 was recorded at 964 ± 55 pA, 1293 ± 63 pA, 490 ± 31 pA, and 452 ± 39 pA, respectively. No significant inhibition was observed by treating the cell with the EO as the amplitude slightly dropped to 812 ± 26 pA, 1095 ± 38 pA, 408 ± 33 pA, and 410 ± 28 pA following the previous order. Nonetheless, the impact of *G. curviflora* EO on AMPAR desensitization and deactivation was tested. The fast amplitude (*τf*), the slow amplitude (*τs*), and the relative amplitude of both (*τf*) and (*τs*) represented by (*af*) were used to attain the weighted tau (*τw*) for AMPAR-current deactivation and desensitization *via* fitting exponentials. The desensitization before *G. curviflora* EO treatment for GluA1, GluA2, GuA1/2, and GluA2/3 was 2.8 ± 0.1 ms, 2.3 ± 0.1 ms, 5.3 ± 0.2 ms, and 2.5 ± 0.3 ms, respectively. After treatment, the desensitization significantly increased to 3.7 ± 0.2 ms, 3.9 ± 0.2 ms, 6.8 ± 0.4 ms, and 3.5 ± 0.3 ms, respectively. The desensitization rate was 0.27, 0.26, 0.15, and 0.28, respectively. Likewise, the same significant effect of oil on AMPAR deactivation rate was observed, whereby the rate dropped from 0.43 ± 0.1, 0.45 ± 0.1, 0.42 ± 0.3, and 0.38 ± 0.2 to 2.6 ± 0.3, 0.24 ± 0.2, 0.21 ± 0.4, and 0.26 ± 0.3 for GluA1, GluA2, GluA1/2, and GluA2/3, respectively.

### 3.3. Antimicrobial Activity

The well diffusion procedure was employed in order to assess *G. curviflora* EO ability to inhibit the growth of *S. aureus*, MRSA, *S. sonnei, E. faecium, E. coli, P. aeruginosa, C. albicans*, and *E. floccosum*. The range of concentration of the prepared *G. curviflora* EO used ranged from 0.025 to 25 *μ*g/mL. The attained results revealed that EO displayed significant antibacterial and antifungal effects against all the tested strains as indicated in Table 2. The EO inhibited the growth of the studied bacteria at concentrations of 0.125–6.25 *μ*L/mL. It inhibited the growth of tested mold (*E. floccosum*) at half concertation required for the inhibition of the yeast *Candida* spp.

### 3.4. Cyclooxygenase Inhibitory Activity

The COX enzymatic inhibition activity of *G. curviflora* EO was accomplished using Cayman Chemical ELISA kit no. 560131. The calculated inhibition percentage of COX-2 for the tested EO revealed inhibition activity of 98.4% and 99.5% at concentrations of 0.25 *μ*g/mL and 0.5 *μ*g/mL, respectively. Similar results were obtained for the inhibition of COX-1, where the inhibition was 98% and 99% for the two tested concentrations, respectively. The results revealed that the *G. curviflora* EO demonstrated a potent but nonselective inhibitory activity towards both COX-2 and COX-1 enzymes.

## 4. Discussion

There are rising interests towards the use of natural biological sources, which are known in general as phytotherapeutic active compounds. The necessity for the search for potential drug candidate from plants is incited by different reasons including the growth of chronic and degenerative diseases, inadequate access to medications, incidents of epidemic antibiotic resistances, and the emergence of new diseases [22–24].

Twenty compounds were identified, accounting for 100% of the total EO, of which 1,6-dimethylspiro[4.5]decane **1**, caryophyllene oxide **2**, and *β*-caryophyllene **3** were the most abundant components representing 27.51%, 20.08%, and 18.28%, respectively. The twenty components could be classified into eight phytochemical groups, of which sesquiterpene hydrocarbon was the most abundant group (38.49%), followed by the oxygenated sesquiterpene (caryophyllene oxide) group (20.08%) and spiroalkane group (29.14%). The major components of sesquiterpene hydrocarbon were *β*-caryophyllene (18.28%), *β*-farnesene (6.2%), and *γ*-caryophyllene (4.95%). The comical compositions of EO of *G. curviflora* growing in Lebanese and Jordanian mountains were reported. Musso et al. reported the identification of 49 compounds in the EO extracted from *G. curviflora* growing in Lebanon, of which 2-isopropyl-5-methyl-3-cyclohexen-1-one (12.51%), (-)-spathulenol (11.73%), *cis*-*Z*-*α*-bisabolene epoxide (8.07%), widdrol (7.0%), (*E*, *Z*)-5, 7-dodecadiene (6.93%), dihydronepetalactone (5.57%), and 4-propyl-cyclohexene (5.43%) were the major constituents [25]. A study conducted by Al-Qudah on EO from fresh and dry aerial parts of *G. curviflora* from Jordan revealed that fresh one was composed mostly of sesquiterpene hydrocarbons (55.27%) whilst air-dried *G. curviflora* was composed mainly of oxygenated monoterpenes (50.31%). The main detected compounds from the fresh sample were *γ*-muurolene (18.54%), 4*aα*,7*α*,6*aα*-nepetalactone (17.76%), *E*-caryophyllene (16.37%), *α*-himachalene (5.63%), *γ*-muurolene (4.30%), and *β*-bisabolene (3.22%), while the major abundant compounds identified in the air-dried sample composed of 4*aα*,7*α*,7*aα*-nepetalactone (43.85%), *E*-caryophyllene (11.53%), and *γ*-muurolene (10.47%) [26].

The qualitative and quantitative variations in the findings could be attributed to the location and collection time and extraction techniques used. In contrast to the previously reported investigation, the mild microwave ultrasonic apparatus for the extraction of the EO was used in the current study. The previously reported studies used the hydrodistillation procedure, which requires reflux for a long time (≥3 h), which can have an impact on the chemical composition of the EO. The plant material used in the current study was collected on the 19th of May, where the previously reported studies worked on a plant collected in April.

Excessive AMPAR activity and upregulation have been implicated with numerous neuropsychiatric disorders and various neurodegenerative diseases such as epilepsy, Parkinson's disease, and ALS [27, 28]. Although the *G. curviflora* EO did not affect the activation of AMPARs, it displayed neuroprotective properties by targeting the receptor's desensitization and deactivation rate. The EO drastically prolonged the state at which the receptor remains in desensitization and deactivation by lowering the rates and consequently reaches a receptor in a nonactive state or a slow current-induced state. The oil showed activity on all tested AMPAR subtypes, yet significantly enhance the desensitization rate in addition to the deactivation rate of the GluA2 subtype comparable to other subtypes. To confirm this finding, we assessed the open state receptors by using 10 mM ligand concentration [3]. While the concentration of the EO was determined through a concentration-dependent curve that defined the plateau level at 80 *μ*M, a further increase in the concentration had no additional effect on desensitization or deactivation. These results inspire a more significant analysis of the *G. curviflora* EO neuroprotective properties. Identification of the active components in the oil is essential in order to understand AMPARs function better and synthesize anticonvulsant drugs targeting AMPARs.

The worldwide evolution of antimicrobial resistance, in addition to the scarcity of novel antibiotic medications, represents a serious public health concern. That means the go-back to a preantibiotic epoch in which infectious diseases caused by multiple-resistant microbial pathogens are uncontrollable [29]. It is well documented that EOs displayed a comparable antimicrobial effect against the tested antibiotic-resistant and antibiotic-susceptible strains; therefore, it could be presumed that the EOs mechanism of action is unlike that from the used antibacterial and antifungal drugs [11]. The antimicrobial activity of the EO extracted from *G. curviflora* leaves and flowers was assessed using broth microdilution assay. The results revealed that the EO affected all screened bacterial and fungal strains. It displayed potent activity against *P. aeruginosa* with a MIC dose of 1.25 *μ*g/mL compared with the positive controls ampicillin and ciprofloxacin, which have MIC values of 25 and 3.12 *μ*g/mL, respectively. *P. aeruginosa* is a Gram-negative bacterium that can cause infection in the respiratory system, urinary tract, wounds, and burns. Furthermore, it can give rise to blood infections causing a series of virulent infectious diseases including diffuse bronchopneumonia, cystic fibrosis, non-CF bronchiectasis, urinary tract catheterization, ecthyma gangrenosum, septic shock, hemorrhage, and necrosis.

The *G. curviflora* EO displayed twofold greater antibacterial activity than that of Ampicillin (positive control) against *S. aureus* with MIC values of 3.12 and 6.25 *μ*g/mL, respectively. On the other hand, it showed twofold weaker anti-MRSA activity than that of ciprofloxacin with MIC values of 25 and 12.5 *μ*g/mL, respectively. Moreover, it exhibited potent anti-*E. coli* activity comparing with ciprofloxacin with MIC values of 1.25 and 0.78 *μ*g/ml, respectively. It showed 1.2-fold lower antibacterial activity against *S. sonnei* as compared with that of ciprofloxacin with MIC values of 14 and 12.5 *μ*g/mL, respectively. The EO revealed anti-*Candida* and antimold activities with MIC values of 6.25 and 3.125 *μ*g/mL as compared with the most potent antifungal drug (fluconazole), which has a MIC dose of 3.12 and 0.66 *μ*g/mL, respectively. *G. curviflora* EO displayed powerful activity against *P. aeruginosa, S. sonnei, E. coli*, *S. aureus*, and MRSA. Our results can be compared with the data reported by Zomorodian et al. on the same strains [30]. They reported that the EO of *N. cataria* displayed a powerful antibacterial activity against Methicillin-Resistant *S. aureus* and *S. sonnei* with MIC values of 0.22 and 2 *μ*l/mL, respectively. Furthermore, a study conducted by Shakeri et al. revealed that EO possesses antibacterial activity against *P. aeruginosa* and *S. aureus* with MIC values of 150 and 14 *μ*g/mL, respectively [31].

Some reports attributed the decent antibacterial activities to the high concentration of bicyclic terpenoid nepetalactone, identified as major compounds of various *Nepeta* species [32, 33]. Furthermore, *in vitro* studies showed that EOs rich in caryophyllene isomers and caryophyllene oxide displayed significant antimicrobial activity, suggesting that the substances possibly participated in this activity [34]. Da Costa et al. found out that *Lantana camara* EO, of which *β*-caryophyllene is the major component (31.5%), exhibited significant antimicrobial activity, especially against *P. vulgaris* (ATCC 13315) and *E. coli* (ATCC 25922) [34]. In agreement with our results, Maia et al. reported that EOs of *Vernonia remotiflora* and *V. brasiliana*, both rich in *β*-caryophyllene (≈40%), inhibited the growth of several tested Gram-negative and Gram-positive bacteria, including *S. aureus* and *P. aeruginosa* [35]. Ghosh et al. [36] evaluated the activity of essential oils of *Alpinia nigra* (47.7 to 49% of *β*-caryophyllene) against Gram-positive and Gram-negative bacteria. The lowest MIC was found against *Yersinia enterocolitica* (1.56 *μ*L/mL).

Cyclooxygenases (COX), known as prostaglandin H_2_ synthases, catalyze the first phase in the biosynthesis of the Nonsteroidal Anti-Inflammatory Drugs (NSAIDs) prostaglandins (PGs) and thromboxanes [37]. Most of the commonly used NSAIDs have various harmful side effects including ringing in the ear, heartburn, stomach pain, hemorrhage, dizziness, headaches, allergic reactions, kidney or liver problems, hyperglycemia, and high blood pressure [38].

The *in vitro* COX inhibitory assay revealed potent inhibitory activity of *G. curviflora* EO as compared with celecoxib, but nonselective towards both COX-1 and COX-2 enzymes with IC_50_ values of 0.069 ± 0.001 and 0.074 ± 0.0015 *μ*g/mL, respectively, which is around 10-fold greater than that of celecoxib (IC_50_ = 0.55 ± 0.0012 *μ*g/mL for COX-1 and 0.6 ± 0.0015 *μ*g/mL for COX-2). Jeppesen et al. reported that EO of *N. parmiriensis* displayed COX-1 inhibitory effect with 45, 85, 75, and 91% inhibition at concentrations of 0.05, 0.5, 5, 50, and 500 *μ*g/mL, respectively, whilst *G. curviflora* EO showed 99% of COX-1 inhibitory action at the 0.5 *μ*g/mL dose which is superior to that of *N. parmiriensis* [39]. Our findings suggest that the EO of *G. curviflora* due to its potent anti-inflammatory potential, which similar to that COX-inhibitory effect of the drug celecoxib that has several adverse reactions [40], could be considered as an alternative option.

## 5. Conclusion

The current investigation explored the chemical ingredients of *G. curviflora* VO, which revealed the presence of twenty phytochemicals, of which 1,6-dimethylspiro[4.5]decane **1**, caryophyllene oxide **2**, and *β*-caryophyllene **3** were the major compounds. The *G. curviflora* VO displayed potent antimicrobial as compared with ciprofloxacin, Ampicillin, and fluconazole. Moreover, it showed potential cyclooxygenase inhibitory activity compared with celecoxib and has potential neuroprotective activity effect. However, further studies in vivo and clinical trial investigations are required to approve these findings and to establish suitable pharmaceutical formulations to be used as formal drugs or food supplements based on the *G. curviflora* VO.

## Figures and Tables

**Table 1:** The chemical composition of *N. curviflora* essential oil.

No.	Compounds	MW (g/mol)	RI	Identification	% area
1	Trans-Carane	138.25	808	MS, RI	0.07

2	*p*-Mentha-3,8-diene	136.23	772	MS, RI	1.58

3	1,3,4-Trimethyl-3-cyclohexenyl-1-carboxaldehyde	152.23	803	MS, RI	0.62

4	Limona ketone	138.21	731	MS, RI	1.58

5	1,6-Dimethylspiro[4.5]decane	166.3	922	MS, RI	27.51

6	Decahydro-3a-methyl-6-methylene-1-(1-methylethyl)cyclobuta[1,2:3,4]dicyclopentadiene	230.39	787	MS, RI	1.97

7	Nepetalactone	166.22	840	MS, RI	5.38

8	cis, trans-1,9-Dimethylspiro[4.5]decane	166.3	875	MS, RI	1.64

9	*β*-Caryophyllene	204.36	924	MS, RI	18.28

10	*β*-Farnesene	204.35	792	MS, RI	6.12

11	*α*-Humulene	204.35	904	MS, RI	1.19

12	Aromadendrene	204.35	917	MS, RI	0.50

13	*β*-Cubebene	204.35	883	MS, RI	2.96

14	Humulen-(v1)	204.35	916	MS, RI	0.17

15	*γ*-Elemene	204.35	837	MS, RI	1.81

16	*γ*-Caryophyllene	204.35	830	MS, RI	4.95

17	*β*-Sesquiphellandrene	204.35	843	MS, RI	2.26

18	Argeol	220.35	734	MS, RI	1.07

19	4-Methylene-2,8,8-trimethyl-2-vinylbicyclo[5.2.0]nonane	204.35	799	MS, RI	0.27

20	Caryophyllene oxide	220.35	732	MS, RI	20.08

	Total (%)				100.00

Grouped components					%

1	Sesquiterpene hydrocarbon				38.49

2	Alcoholic sesquiterpenoid				1.07

3	Oxide sesquiterpenoid				20.08

4	Monoterpene hydrocarbon				1.65

5	Aldehydic monoterpenoid				0.62

6	Ketonic monoterpenoid				1.97

7	Monoterpenoid lactone				5.38

8	Spiroalkane				29.14

9	Ketone				1.58

	Total identified groups (%)				100.00

MW: molecular weight; RI: retention index; and RT: retention time.

**Table 2:** Antimicrobial activities (MICs) of *G. curviflora* EO (*μ*g/mL).

Microorganisms	*G. curviflora* EO	Ampicillin	Ciprofloxacin	Fluconazole
*S. aureus*	3.12	6.25	0.78	0
MRSA	25	0	12.5	0
*S. sonnei*	14	20	12.5	0
*E. faecium*	6.25	1.56	0.78	0
*E. coli*	1.25	3.12	0.78	0
*P. aeruginosa*	1.25	0	3.12	0
*C. albicans*	6.25	0	0	3.12
*E. floccosum*	3.12	0	0	0.66

## Data Availability

All the utilized data to support the findings of the current study are included in the article.

## References

[1] Egbuna C., Kumar S., Ifemeje J. C., Ezzat S. M., Kaliyaperumal S. (2019). *Phytochemicals as Lead Compounds for New Drug Discovery*.

[2] Tanveer M., Wagner C., Ul Haq M. I. (2020). Spicing up gastrointestinal health with dietary essential oils. *Phytochemistry Reviews*.

[3] Qneibi M., Hamed O., Fares O. (2019). The inhibitory role of curcumin derivatives on AMPA receptor subunits and their effect on the gating biophysical properties. *European Journal of Pharmaceutical Sciences*.

[4] Li B., Webster T. J. (2018). Bacteria antibiotic resistance: new challenges and opportunities for implant-associated orthopedic infections. *Journal Orthopaedic Research*.

[5] Jaradat N., Al-lahham S., Abualhasan M. N. (2018). Chemical constituents, antioxidant, cyclooxygenase inhibitor, and cytotoxic activities of *Teucrium pruinosum* boiss. Essential oil. *BioMed Research International*.

[6] Suleyman H., Demircan B., Karagoz Y. (2007). Anti-inflammatory and side effects of cyclo-oxygenase inhibitors. *Pharmacological Reports*.

[7] Al-Bakri A. G., Afifi F. U. (2007). Evaluation of antimicrobial activity of selected plant extracts by rapid XTT colorimetry and bacterial enumeration. *Journal of Microbiological Methods*.

[8] Ali A., Tabanca N., Demirci B. (2016). Chemical composition and biological activity of essential oils from four *Nepeta* species and hybrids against *Aedes aegypti* (L.) (Diptera: Culicidae). *Records Natural Product*.

[9] Sharma A., Cannoo D. S. (2013). Phytochemical composition of essential oils isolated from different species of genus *Nepeta* of Labiatae family: a review. *Pharmacophore*.

[10] Jaradat N. A., Al-lahham S., Zaid A. N. (2019). *Carlina curetum* plant phytoconstituents, enzymes inhibitory and cytotoxic activity on cervical epithelial carcinoma and colon cancer cell lines. *European Journal of Integrative Medicine*.

[11] Shehadeh M., Jaradat N., Al-Masri M. (2019). Rapid, cost-effective and organic solvent-free production of biologically active essential oil from Mediterranean wild *Origanum syriacum*. *Saudi Pharmaceutical Journal*.

[12] Vinaixa M., Schymanski E. L., Neumann S., Navarro M., Salek R. M., Yanes O. (2016). Mass spectral databases for LC/MS- and GC/MS-based metabolomics: state of the field and future prospects. *TrAC Trends in Analytical Chemistry*.

[13] Wei X., Koo I., Kim S., Zhang X. (2014). Compound identification in GC-MS by simultaneously evaluating the mass spectrum and retention index. *The Analyst*.

[14] Qneibi M., Hamed O., Natsheh A.-R. (2019). Inhibition and assessment of the biophysical gating properties of GluA2 and GluA2/A3 AMPA receptors using curcumin derivatives. *PLoS One*.

[15] Qneibi M., Jaradat N., Emwas N. (2019). Effect of geraniol and citronellol essential oils on the biophysical gating properties of AMPA receptors. *Applied Sciences*.

[16] Qneibi M., Jaradat N., Hawash M. (2019). The neuroprotective role of *Origanum syriacum* L. and *Lavandula dentata* L. essential oils through their effects on AMPA receptors. *BioMed Research International*.

[17] Forbes B. A., Sahm D. F., Weissfeld A. S. (2007). *Study Guide for Bailey & Scott’s Diagnostic Microbiology*.

[18] Wikler M. A. (2007). *Performance Standards for Antimicrobial Susceptibility Testing: Seventeenth Informational Supplement*.

[19] Forbes B. A., Sahm D. F., Weissfeld A. S. (2016). *Study Guide for Bailey and Scott’s Diagnostic Microbiology*.

[20] Klepser M. E., Wolfe E. J., Jones R. N., Nightingale C. H., Pfaller M. A. (1997). Antifungal pharmacodynamic characteristics of fluconazole and amphotericin B tested against Candida albicans. *Antimicrobial Agents and Chemotherapy*.

[21] Falahati M., Tabrizib N. O., Jahaniani F. (2005). Anti dermatophyte activities of *Eucalyptus camaldulensis* in comparison with Griseofulvin. *Iranian Journal of Pharmacology & Therapeutics*.

[22] Ramawat K., Dass S., Mathur M. (2009). *The Chemical Diversity of Bioactive Molecules and Therapeutic Potential of Medicinal Plants, Herbal Drugs: Ethnomedicine to Modern Medicine*.

[23] Reid A.-M., Oosthuizen C. B., Fibrich B. D. (2018). *Traditional Medicine: The Ancient Roots of Modern Practice, Medicinal Plants for Holistic Health and Well-Being*.

[24] Srivastava A., Srivastava P., Pandey A., Khanna V., Pant A. (2019). *Phytomedicine: A Potential Alternative Medicine in Controlling Neurological Disorders, New Look to Phytomedicine*.

[25] Musso L., Scaglia B., Haj G. A. (2017). Chemical characterization and nematicidal activity of the essential oil of *Nepeta nuda* L. ssp. pubescens and *Nepeta curviflora* Boiss. from Lebanon. *Journal of Essential Oil Bearing Plants*.

[26] Al-Qudah M. A. (2016). Antioxidant acitvity and chemical composition of essential oils of fresh and air-dried Jordanian *Nepeta curviflora* Boiss. *Journal of Biologically Active Products from Nature*.

[27] Mousa S. S., Dikshit M. (2004). AMPA receptor regulation mechanisms: future target for safer neuroprotective drugs. *International Journal of Neuroscience*.

[28] Kwak S., Weiss J. H. (2006). Calcium-permeable AMPA channels in neurodegenerative disease and ischemia. *Current Opinion in Neurobiology*.

[29] Jaradat N., Shawarb N., Hussein F. (2018). Antibacterial and antioxidant screening of semi-synthetic naringin based hydrazone and oxime derivatives. *Jundishapur Journal Microbiology*.

[30] Zomorodian K., Saharkhiz M. J., Shariati S., Pakshir K., Rahimi M. J., Khashei R. (2012). Chemical composition and antimicrobial activities of essential oils from *Nepeta cataria* L. Against common causes of food-borne infections. *ISRN Pharmaceutics*.

[31] Shakeri A., Khakdan F., Soheili V., Sahebkar A., Rassam G., Asili J. (2014). Chemical composition, antibacterial activity, and cytotoxicity of essential oil from *Nepeta ucrainica* L. spp. kopetdaghensis. *Industrial Crops and Products*.

[32] Bourrel C., Perineau F., Michel G., Bessiere J. M. (1993). Catnip (*Nepeta cataria* L.) essential oil: analysis of chemical constituents, bacteriostatic and fungistatic properties. *Journal of Essential Oil Research*.

[33] Zenasni L., Bouidida H., Hancali A. (2008). The essentials oils and antimicrobial activity of four Nepeta species from Morocco. *Journal Medicinal Plant Research*.

[34] Costa J. G. M. D., Sousa E. O. D., Rodrigues F. F. G., Lima S. G. D., Braz-Filho R. (2009). Composição química e avaliação das atividades antibacteriana e de toxicidade dos óleos essenciais de Lantana camara L. e Lantana sp. *Revista Brasileira de Farmacognosia*.

[35] Maia A. I. V., Torres M. C. M., Pessoa O. D. L. (2010). Óleos essenciais das folhas de Vernonia Remotiflora e Vernonia Brasiliana: composição química e atividade biológica. *Química Nova*.

[36] Ghosh S., Ozek T., Tabanca N. (2014). Chemical composition and bioactivity studies of *Alpinia nigra* essential oils. *Industrial Crops and Products*.

[37] Jaradat N., Al-Maharik N. (2019). Fingerprinting, antimicrobial, antioxidant, anticancer, cyclooxygenase and metabolic enzymes inhibitory characteristic evaluations of stachys viticina boiss. Essential oil. *Molecules*.

[38] Moore N. (2020). Coronary risks associated with diclofenac and other NSAIDs: an update. *Drug Safety*.

[39] Jeppesen A. S., Soelberg J., Jäger A. K. (2012). Antibacterial and COX-1 inhibitory effect of medicinal plants from the pamir mountains, Afghanistan. *Plants*.

[40] Levy M. B., Fink J. N. (2001). Anaphylaxis to celecoxib. *Annals of Allergy, Asthma & Immunology*.

